# A Stir about Female Physicians

**Published:** 1860-12

**Authors:** 


### A Stir about Female Physicians.

The Pennsylvania State Medical Society, and Philadelphia County Society have passed resolutions refusing their members consultation with Female Physicians. The Montgomery County Medical Society of the same State objects to the course taken relative to Medical Sisters, by those Societies. Dr. B. Dowler, of the New Orleans Medical & Surgical Journal, in an article of some length, advocates the female side of the question dividing the profession in Pennsylvania. He says: "As there is no sex in science so there is no ethical code by which competent female physicians must be excluded from the pale of the profession solely on account of their gender." We think this matter comes more properly before Society in general. If public opinion tolerates the practice of medicine in a female, and decides that *all* the duties of a physician will comport with the proper grace, modesty and gentle bearing of females, it is all that is necessary to give them a place in the profession. As a member of social society we say that when females seek the position of physicians they leave the sphere of greatness nature designed them to move in, and in which society desires them to remain. As a medical man we look to the capability, honesty and uprightness of those who ask admission into the profession. We say let women doctors *alone* and they will soon learn the error of their way, if an error it be, and return to their *domestic* and literary greatness.

